# Ras suppressor-1 (RSU-1) promotes cell invasion in aggressive glioma cells and inhibits it in non-aggressive cells through STAT6 phospho-regulation

**DOI:** 10.1038/s41598-019-44200-8

**Published:** 2019-05-23

**Authors:** Maria Louca, Andreas Stylianou, Angeliki Minia, Vaia Pliaka, Leonidas G. Alexopoulos, Vasiliki Gkretsi, Triantafyllos Stylianopoulos

**Affiliations:** 1 Cancer Biophysics Laboratory, Department of Mechanical and Manufacturing Engineering, University of Cyprus, Nicosia, Cyprus; 2 ProtATonce Ltd., Athens, Greece; 3 Department of Mechanical Engineering, National Technical University of Athens, Athens, Greece; 4 Biomedical Sciences Program, Department of Life Sciences, School of Sciences, European University Cyprus, Nicosia, Cyprus

**Keywords:** CNS cancer, Metastasis

## Abstract

Most gliomas are invasive tumors formed from glial cells and associated with high mortality rates. In this study, we characterized four glioma cell lines of varying degree of aggressiveness (H4, SW1088, A172 and U87-MG) in terms of morphology, cytoskeleton organization and stiffness, and evaluated their invasive potential by performing invasion, colony forming and spheroid invasion assays. Cells were divided into two distinct groups: aggressive cell lines (A172 and U87-MG) with more elongated, softer and highly invasive cells and less aggressive cells (H4 and SW088). Interestingly, we found that Ras Suppressor-1 (RSU-1), a cell-matrix adhesion protein involved in cancer cell invasion, was significantly upregulated in more aggressive glioma cells compared to less aggressive. Importantly, *RSU-1* silencing had opposing effects on glioma cell invasion depending on their aggressiveness, inhibiting migration and invasion of aggressive cells and promoting those of less aggressive cells. Finally, we found that *RSU-1* silencing in aggressive cells led to decreased Signal Transducer and Activator of Transcription6 (STAT6) phosphorylation and Matrix Metalloproteinase13 (MMP13) expression in contrast to less invasive cells. Our study demonstrates that *RSU-1* promotes invasion of aggressive glioma cells and inhibits it in the non-aggressive cells, indicating that it could serve as a predictor of gliomas progression.

## Introduction

Gliomas are characterized by rapid progression and high mortality rates. Glioblastoma multiforme (GBM), a type of astrocytoma arising from uncontrolled proliferation of astrocytes, is the most aggressive type of malignant brain tumors in the cerebral hemispheres^1,2^. Its treatment, as with all gliomas, is multimodal consisting of surgical removal, radiotherapy and chemotherapy. However, although there are several therapeutic modalities, GBM still has poor prognosis due to the increased incidence of invasion of cancer cells into adjacent tissue forming metastases^3–5^. Indeed, invasion and migration of glioma cells away from the main tumor mass, is one of the most important issues in glioma therapy, being highly associated with decreased survival rates^6^. Genetic instability and deregulation of multiple focal adhesion (FA) proteins are known to be crucially involved in these processes^7,8^. FA proteins are localized at cell-extracellular matrix (ECM) sites transducing biochemical and biomechanical cues from the ECM and integrins to effector molecules inside the cell, while most of them are directly interacting with actin cytoskeleton and are involved in many cellular processes such as survival, differentiation, proliferation, migration and invasion^9,10^.

Ras Suppressor-1 (RSU-1) was first identified as a suppressor of *Ras* oncogene^11^, exhibiting growth suppression effects^12–16^ while it was later found to be localized to cell-ECM adhesion sites through its interaction with Particularly Interesting New Cysteine-Histidine rich protein (PINCH-1)^17^. Beyond cancer cell proliferation, RSU-1 has been also documented to play a crucial role in cancer cell migration and invasion^18–22^ both of which are fundamental steps in the metastatic process. Little is known, however, regarding *RSU-1* expression and its role in tumors of the central nervous system^23^. It is hypothesized though that it should be involved in glioma pathogenesis as well, as it seems to play a critical role in regulating synapse maturation by preventing spontaneous clustering of extrasynaptic acetylocholine receptors^24^ and enhances Nerve Growth Factor (NGF)-induced neuronal differentiation^25^. Also, lack of *RSU-1* activates c-Jun N-terminal protein kinase (JNK) and neural stem and progenitor cell (NSPC) proliferation^26^. Hence, the main objective of this research work was the *in vitro* characterization of a panel of four commercially available glioma cell lines of varying degrees of invasiveness, namely H4, SW1088, A172 and U87-MG in terms of morphology, cytoskeleton organization, stiffness and aggressiveness as well as the determination of the involvement of RSU-1 in the metastatic properties of glioma cells.

## Materials and Methods

### Glioma cell lines

A panel of human glioma cell lines (H4, SW1088, A172 and U87-MG) was purchased from ATCC. H4 cells are non-tumorigenic epithelial brain cells, SW1088 are responsible for astrocytoma formation, whereas U87-MG and A172 were isolated from patients with GBM. Cells were grown in high-glucose DMEM medium supplemented with 10% fetal bovine serum and 1% antibiotic/antimycotic and were cultured in a humidified incubator supplied with 5% CO2 at 37 °C.

### Antibodies and reagents

Anti-RSU-1 rabbit polyclonal antibody for immunoblotting was kindly provided by Dr. Mary Lou Cutler, Professor at the Uniformed Services University of the Health Sciences, Bethesda USA. Anti-pSTAT6 and anti-STAT6 were obtained from Cell Signaling. Anti-MMP13 was purchased from Abcam. Phospho-STAT6 inhibitor, AS1517499, was obtained from Axon Medchem. *RSU-1* siRNA was purchased from Santa Cruz Biotechnology. Rhodamine-Phalloidin was obtained from Biotium and 4′,6-Diamidino-2-Phenylindole (DAPI) was obtained from Roche. Transwell inserts were purchased from Greiner Bio-One and Matrigel as well as Collagen I was obtained from Corning. QIAzol Lysis Reagent was purchased from QIAGEN.

### Cell Elongation and Factor E measurement

Pictures of individual live cells were taken using a Nikon Eclipse TS100 inverted microscope equipped with a digital camera and a Nikon Ph1 DL 10x 0.25 phase microscope objective lens. ImageJ software was used to measure the factor E of the cells, which is calculated by dividing the longest axis by the shortest axis and subtracting one^27^. The elongation factor E describes the extent to which the equimomental ellipse is lengthened or stretched out^28^. Given the fact that factor E is zero (0) for a circle, and one (1) for an ellipsoid with an axis ratio 1:2, E values between 0–0.5 are considered to correspond to spherical cells, 0.5–1 to ellipsoids, and E values higher than 1 are considered to correspond to elongated cells^29^.

### Atomic Force Microscopy (AFM)

Cells were cultured in 35 mm petri dishes overnight. Then the samples were directly mounded on AFM sample plates. The Young’s modulus of cells was acquired by using a Molecular Imaging-Agilent PicoPlus AFM system with silicon nitride probes and a round, ball-shape tip (CP-PNPL-BSG-A-5, sQube, 5 μm diameter spheres, spring constant of 0.08 N/m). The Young’s modulus which is in essence the stiffness of live cells was assessed by acquiring 8 × 8 points of force curves in an area of 5 × 5 μm near the center of the cells^20^. For the acquisition of the force-displacement curves a set point of 1nN normal force at a 2 μm/s strain rate on each of the studied cells was used^30^. Subsequently, the AtomicJ software^31^ and the Hertz model were employed for the calculation of the Young’s modulus, while for the calculations the Poisson’s ratio was assumed to be equal to 0.5.

### Cytoskeleton assay staining and morphology identification

H4, SW1088, A172 and U87 cells were plated at a density of 10,000 cells per well on glass coverslips coated with 0.1% gelatin. Twenty-four (24) hours later, cells were fixed with 4% PFA for 20 min and they were then permeabilized using a buffer containing 0.1% Triton X-100 and 2 mg/ml BSA in PBS. Cells were finally double stained with Rhodamine phalloidin and DAPI^32–34^. In order to characterize the actin cytoskeleton in terms of stress fiber formation and orientation, the freeware tool FilamentSensor was used. This tool is an open source software written in Java [http://filament-sensor.de/], for semi-automated detection of line segments in images. It is primarily designed for detection of actin fibers from microscopy images and it is based on the filament sensor (FS), a fast and robust processing sequence which detects and records location, orientation, length, and width for each single filament of an image^32^. In the analyzed figures the different fiber orientations of the F-actin stress fibers were represented with a different color^30,32^.

### Transwell migration and invasion assays

Cell migration and invasion assays were performed using transwell chambers with 8 μm pore size membranes. The membranes of the inserts were either left uncoated and used for migration or were coated with diluted Matrigel (1:20) 24 h before cell seeding and used for invasion^35,36^. In total, 3.5 × 10^4^ cells in 0.5 ml of serum-free medium were added to the upper chamber, while the lower chamber was filled with 750 μl of culture medium supplemented with 10% fetal bovine serum and 1% antibiotic/antimycotic. After a 24 h incubation, the non-invading cells were removed from the upper surface of the membrane using a cotton swab. The cells that had passed through the membrane were fixed with 4% PFA for 20 min, and stained with 0.1% crystal violet in PBS for 20min^37–39^. For quantification, cells in nine selected microscopic fields per well were counted, and the sum was calculated. Three independent experiments were performed.

### Tumor spheroids formation in collagen gels

The “hanging drop” technique was used to generate tumor spheroids, as described previously^20^. A suspension of 2.5 × 10^4^ glioma cells (H4, SW1088, A172 and U87-MG) was prepared and several drops of 20 μl each containing 500 cells were placed on the cover of a culture dish. Spheroids were allowed to grow for 24 hours^40–42^. Individual spheroids were then embedded in wells of a 96-well plate containing 1.0 mg/ml collagen I^20^. Pictures were taken at time zero and at several time points. Spheroid’s size was determined using the ImageJ software, and taking the mean length of the major and minor axis of the spheroid at a given time point compared to the initial size at time zero.

### Phospho-STAT6 inhibitor treatment

Cells were treated with 100, 200 or 300 nM of phospho-STAT6 inhibitor, AS1517499, for at least 48 h in high-glucose DMEM medium supplemented with 10% fetal bovine serum and 1% antibiotic/antimycotic according to previous studies^43,44^. Inhibition of STAT-6 phosphorylation was verified by immunoblotting following standard procedures.

### Transfection with siRNA

All cells were transfected with 100 nM siRNA against RSU-1 or a non-specific control siRNA, using the HiPerfect reagent (Qiagen) according to the manufacturer’s guidelines. Cells were harvested 48 h post-transfection.

### Soft agar growth assay

Cells were trypsinized, suspended at a concentration of approximately 5 × 10^3^ cells/ml in 0.3% soft agar and placed on a layer of 0.6% soft agar in a six-well plate. After a 30 day incubation in a humidified atmosphere in the presence of 5% CO_2_ at 37 °C, colonies were formed and were subsequently fixed with 4% PFA and stained with 0.01% crystal violet in PBS for 1 h^45,46^. Colonies were examined using an inverted microscope. Five randomly selected microscopic fields per well were used for quantification, and the number of total colonies per well were counted. Also, ImageJ software was used to measure the size of the colonies (area). The experiment was performed in triplicate for each cell line.

### Sample preparation and phosphoproteins’ measurements

A custom 21-plex assay was built aiming to investigate cell invasion through the regulation of influential phosphoproteins. The Multiplex assay was designed following literature search to detect the most influential phosphoproteins and discover if these signaling molecules play an important role in glioma cell invasion upon RSU-1 silencing. Cells were lysed using cell lysis buffer (LysisPlex, ProtATonce, Cat Nr: LPA01) 48 h post-transfection with appropriate siRNA (NSC or RSU-1 siRNA) and protein concentration was adjusted to 200 μg/ml with lysis buffer. Cell lysates were used for the phosphoprotein measurements. Twenty-one (21) capture antibodies coupled to Luminex magnetic beads and 21 biotinylated detection antibodies were multiplexed to create the bead mix and the detection mix, respectively. The coupled beads (50 μl of the bead mix) were incubated with the samples on a flat bottom 96-well plate on a shaker at 900 rpm for 90 minutes at room temperature. Then, detection mix was added, and the samples were incubated further on a shaker at 900 rpm for 60 minutes at room tempserature. The final step was the addition of freshly prepared SAPE solution (Streptavidin, R-Phycoerythrin conjugate, Cat Nr: S866, Invitrogen) for the detection of the signal. Following a 15 minute incubation time with SAPE, samples were measured with the Luminex FlexMAP 3D instrument.

The following phospho-proteins were assessed: Transcription factor p65 (NF-Κb/TF-65, Cat Nr: P-NFKB-A01), Mitogen-activated protein kinase 12 (p38, Cat Nr: P-MK12-A01), RAC-alpha serine/threonine-protein kinase (AKT1, Cat Nr: P-AKT1-01), Serine/threonine-protein kinase WNK1 (WNK1, Cat Nr: P-WNK1-A01), Tyrosine-protein phosphatase non-receptor type 11 (PTN11, Cat Nr: P-PTN11-A01), Signal transducer and activator of transcription 3 (STAT3, Cat Nr: P-STAT3-A01), Heat shock protein beta-1 (HSP27/HSPB1, Cat Nr: P-HSPB1-A01), Transcription factor AP-1 (JUN, Cat Nr: P-JUN-A01), Signal transducer and activator of transcription 5 A (STAT5, Cat Nr: -P-STAT5-A01), Glycogen synthase kinase-3 alpha/beta (GSK3A/B, Cat Nr: P-GSK3A/B-A01), 40 S ribosomal protein S6 (RS6, Cat Nr: P-RS6-A01), Ribosomal protein S6 kinase beta-1 (p70S6K, Cat Nr: p-KS6B1-A01), Platelet-derived growth factor receptor beta (PGFRb, Cat Nr: P-PDGFRb-A01), Tyrosine-protein kinase Lck (LCK, Cat Nr: P-LCK-A01), Ribosomal pro/, tein S6 kinase alpha-1 (RSK1, Cat Nr: P-KS6A1-A01), Nuclear factor erythroid 2-related factor 2 (NRF2, Cat Nr: P-NRF2-A01), Cyclic AMP-responsive element-binding protein 1 (CREB1, Cat Nr: P-CREB1-A01), Signal transducer and activator of transcription 6 (STAT6, Cat Nr: P-STAT6-A01), Focal adhesion kinase 1 (FAK1, Cat Nr: P-FAK1-A01), Proto-oncogene tyrosine-protein kinase SRC (SRC, Cat Nr: P-SRC-A01), NF-kappa-B inhibitor alpha (Ik-Ba, Cat Nr: P-NFkB-A01), Proline-rich AKT1 substrate 1 (AKTS1, Cat Nr: P-AKTS1-A01).

### RNA isolation and Real-Time Polymerase Chain Reaction (RT-PCR)

Τotal RNA was extracted from cells using QIAzol Lysis Reagent and quantification of gene expression was assessed by RT-PCR using the ΔΔCt method as described previously^47^. The sequences of the specific primers are described in Table 1.

**Table 1:** Primer sequences used for RT-PCR.

Primer name	Sequence
RSU-1	Forward 5′ AGGCCACAGAGCAAGGTCTA 3′
	Reverse 5′ CGT GCA ATC TCA AAA GCT CA 3′
MMP13	Forward 5′ TGGCATTGCTGACATCATGA3′
	Reverse 5′ GCCAGAGGGCCCATCAA3′
β-actin	Forward 5′ CGAGCACAGAGCCTCGCCTTTGCC-3′
	Reverse 5′ TGTCGACGACGAGCGCGGCGATAT-3′

### Protein extraction and western blotting

Whole cell extracts were prepared using radio immunoprecipitation assay (RIPA) buffer containing a protease inhibitor cocktail tablet (Sigma) and western blot analysis was performed using standard immunoblotting protocols as described previously^20,47^. The detection of the antibody was done with enhanced chemiluminescent system from Pierce and Kodak Biomax light films or using ChemiDoc XRS+ Imaging System (BioRad) and protein expression was quantified compared to the β-actin loading control using the ImageJ software. The mean intensity of respective protein bands from four different immunoblots was used for the quantification, as indicated.

### Statistical analysis

Results are represented as mean ± standard error (SE). Significant changes were determined by Student’s t test using two-tail distribution. Differences with p values < 0.05 were considered as statistically significant (indicated by an asterisk *).

## Results

### Glioma cell morphology is associated with their invasive behavior

**Figure 1:**
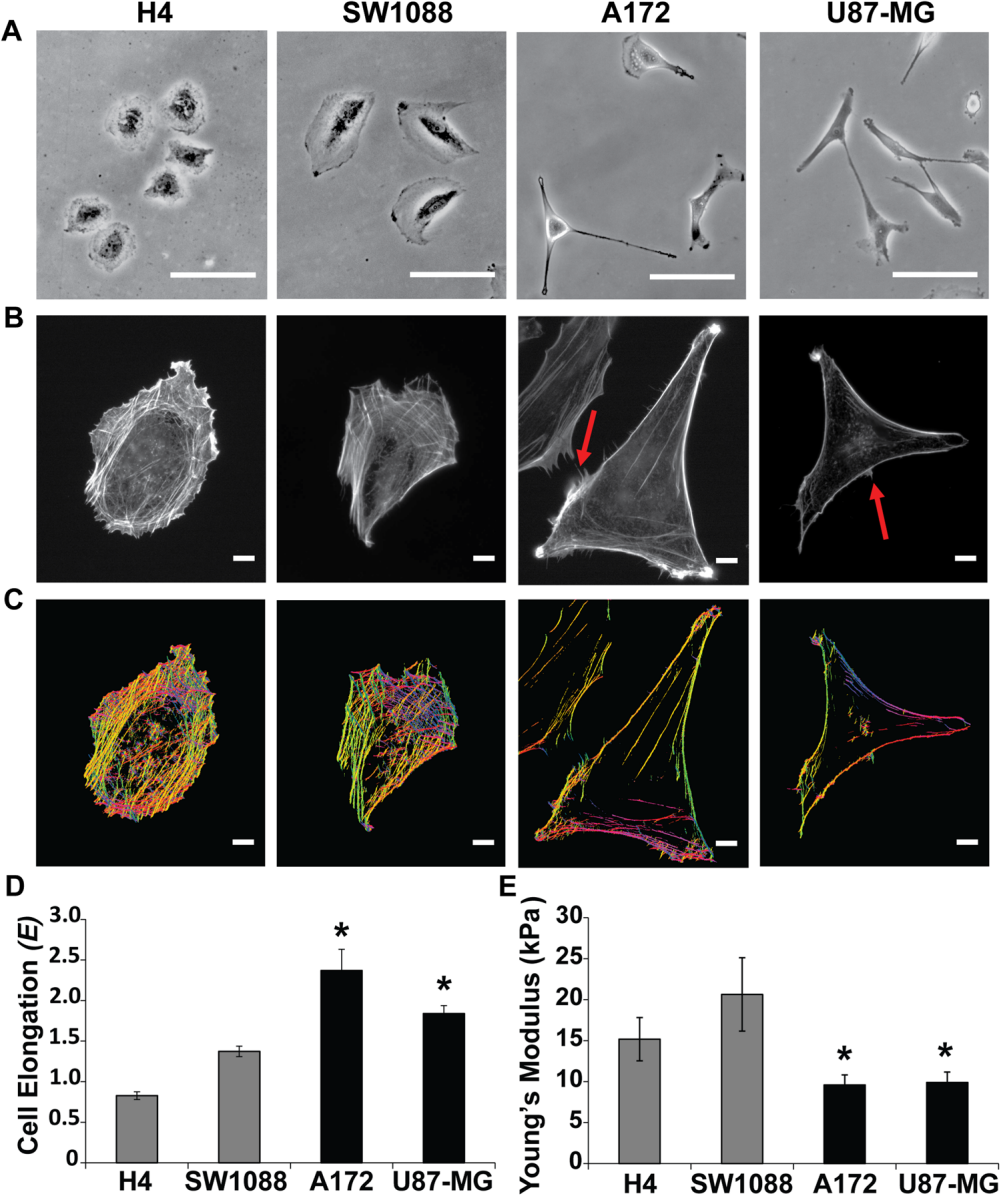
Morphological characterization of glioma cells. Representative images from **(A)** optical microscope imaging of H4, SW1088, A172 and U87-MG cells. Scale bar: 100 μm **(B)** fluorescence microscope imaging of phalloidin-stained cells and **(C)** stress fiber orientation analysis using the FilamentSensor tool software where each color matches to a different fiber orientation (n = 30 from each cell line and each group). Scale bar: 10 μm **(D)** Cells elongation quantification, factor E was calculated from optical microscopy images of live cells, and **(E)** Young’s modulus measurements using AFM. Asterisks denote a statistically significant difference (p < 0.05) compared to H4 data.

We first set out to characterize the four human glioma cell lines (H4, SW1088, A172 and U87-MG) with regard to their morphology and assess the possible connection that morphology may have with cell aggressiveness. Optical microscopy imaging and elongation analysis demonstrated that A172 and U87-MG cells, which cause GBM, were more elongated than H4 and SW1088, which are non-tumorigenic epithelial and fibroblast-like cells, respectively (Fig. 1A,D)^48–50^. As cytoskeletal remodeling is fundamental for metastasis-related processes, such as migration and invasion^51^, cells were also stained with phalloidin, a widely-used fungal toxin known to bind filamentous actin, in order to detect possible changes in the organization of the cytoskeleton. Figure 1B shows that H4 and SW1088 cells exhibited abundant F-actin stress fibers in contrast to A172 and U87-MG cells where filamentous actin was not that prominent. Furthermore, we used the FilamentSensor tool software^32^ in order to investigate the stress fiber orientation in each cell line. As shown in Fig. 1C, stress fibers in H4 and SW1088 cells presented random orientation, while A172 and U87-MG exhibited well-organized actin fibers. Interestingly, A172 and U87-MG cells formed more lamellipodia, the thin sheet-like membrane protrusions found at the leading edge of migrating cells, as indicated by the arrow in Fig. 1B, suggesting that these two cell lines are more prone to migration.

### Elongated and softer glioma cells are more invasive

Intrigued by the finding that A172 and U87-MG have morphological characteristics that differentiate them from H4 and SW1088, we sought to find out whether their stiffness was also related to their invasive potential. Several studies in the literature have connected these two, showing that the softer the cell the more likely it is to exhibit malignant characteristics^30^. For this purpose, AFM was used to measure cell stiffness of the four glioma cell lines. We found that A172 and U87-MG cells were softer than H4 and SW1088 (Fig. 1E), as demonstrated by the reduced Young’s modulus value. The absolute values of the cell’s Young’s modulus were found to be H4:15.2 ± 2.6 kPa, SW1088:20.6 ± 4.5 kPa, A172:9.6 ± 1.2 kPa and U87-MG:9.9 ± 1.3 kPa (Young’s Modulus Absolute Value = Average ± Standard Error), which lie within the expected values for live cells^52,53^.

**Figure 2:**
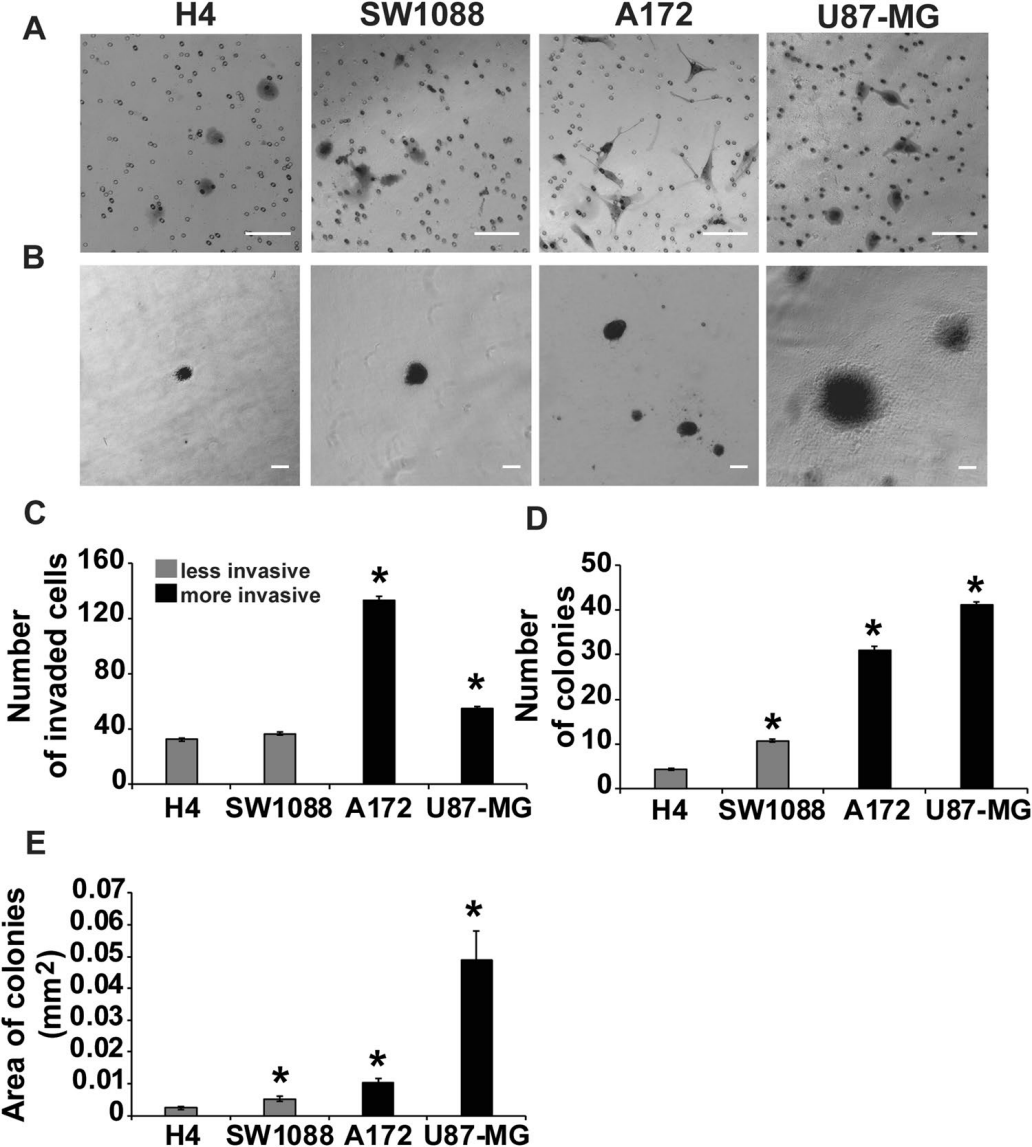
Aggressiveness of glioma cells. (**A**) Representative images of a transwell invasion assay using matrigel-coated inserts. The experiment was performed for 24 h and the invading cells were counted in nine (9) randomly chosen microscopic fields per transwell. **(B)** Representative images of soft agar assay for 30 days. For quantitative analysis, five (5) images per well were taken with inverted microscope. Scale bar:100 μm. **(C)** Mean of total number of invaded cells per transwell of cell invasion compared to H4 cell line. Each sample was run in triplicate and three (3) independent experiments were performed. **(D)** Mean of total number of colonies per well compared to H4 cell line. Each sample was run in triplicate. **(E)** Area of colonies in mm^2^ in average per well. Asterisks denote a statistically significant difference (p < 0.05) compared to H4 data.

To test our hypothesis that cell stiffness is related to malignant characteristics, such as cell migration and invasion, a transwell invasion assay was performed. Over a 24-h period, the number of cells that invaded through matrigel differed among the four glioma cell lines (Fig. 2A,C). The total number of cells invading through matrigel in average per transwell is presented in Fig. 2C. The more aggressive U87-MG and A172 cells showed a statistically significant increase in invasion compared to the less aggressive H4 and SW1088.

To corroborate the data obtained from the transwell invasion assay, glioma cancer cell spheroids were generated from all four cell lines under study and embedded in 1 mg/ml collagen I gels, as described previously^20^. Notably, the rate of tumor spheroid invasion was dramatically different between the cell lines, further verifying that A172 and U87-MG are more invasive than H4 and SW1088. Supplementary Fig. 1A shows representative images of H4 and A172 spheroids at their corresponding times. Specifically, A172 and U87-MG tumor spheroids invaded quickly and started dissociating from the original spheroid mass within 6 h, while H4 spheroids reached a similar state at 16 h, and SW1088 spheroids at 12 h (Supplementary Fig. 1B). Thus, the incubation time needed for spheroid invasion was correlated with the aggressiveness of cells and their invasive potential.

### Aggressive cells construct colonies in unfavorable conditions

As our findings indicated that A172 and U87-MG cells were more elongated, softer, and formed tumor spheroids that invaded faster through collagen gels than H4 and SW1088 cells, we next examined the degree of their aggressiveness using the standard soft agar assay (Fig. 2B). The number of colonies formed on soft agar by the four glioma cell lines at the end of the 30-day period was measured and results are shown in Fig. 2D. U87-MG and A172 cells formed multiple large colonies on agar, while the other two cell lines only formed a few small colonies (Fig. 2E). Notably, U87-MG cells formed colonies of the largest size. These results further confirm that A172 and U87-MG exhibit a more invasive phenotype than H4 and SW1088.

### RSU-1 protein and mRNA expressions are elevated in the aggressive glioma cell lines

**Figure 3:**
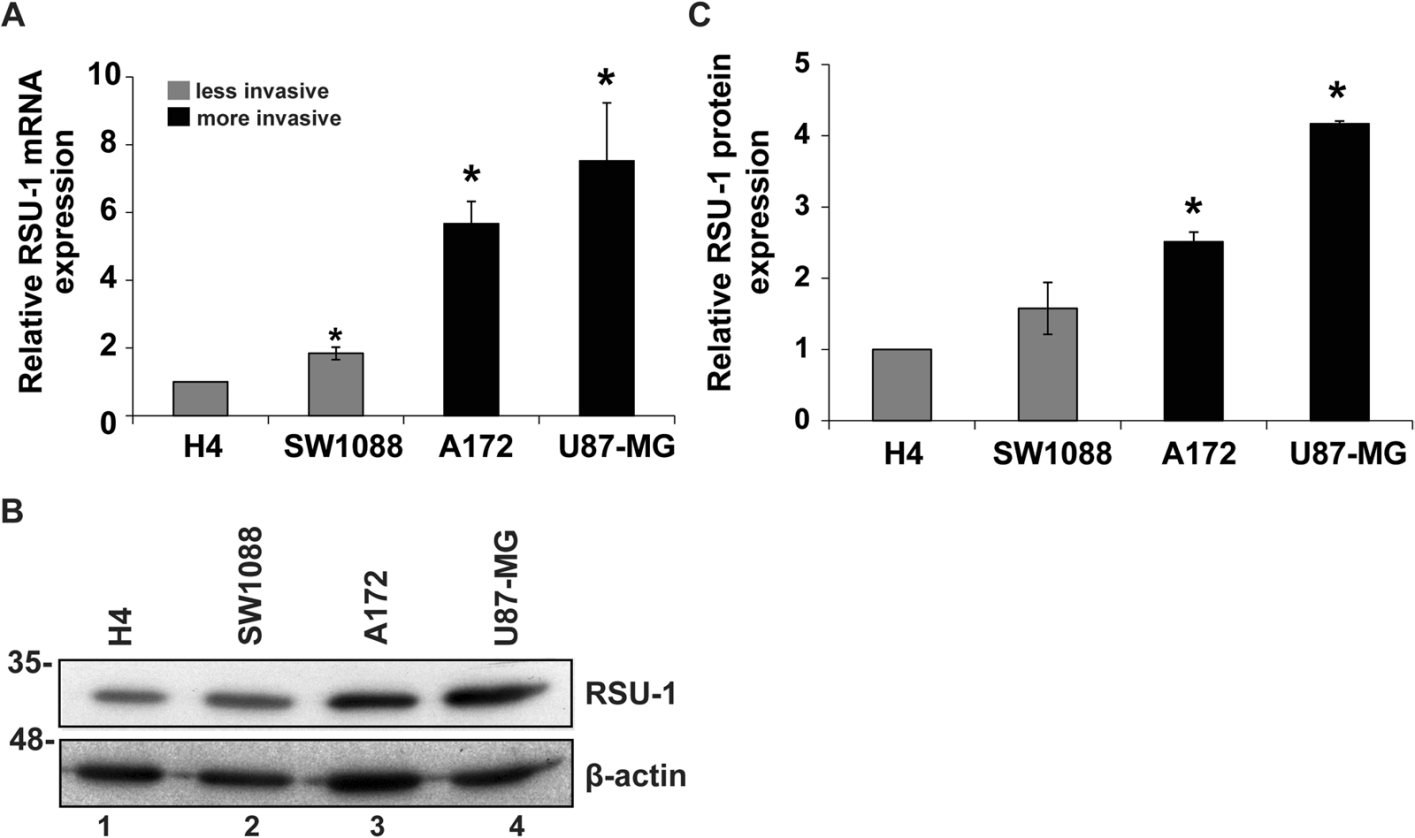
RSU-1 expression is elevated in more invasive glioma cells. (**A)** Relative *RSU-1* mRNA expression for the four glioma cell lines under study. Four independent Real Time PCR experiments were performed, and data were analyzed using the ΔΔCt method. **(B)** Western blot for *RSU-1* protein expression, using β-actin as a loading control and H4 as a sample control. Cropped blots are from samples run on the same gel and original pictures of the western blots are displayed in Supplementary Fig. 2. **(C)** Graph shows the quantification of *RSU-1* protein expression by ImageJ software from three different western blots. Asterisks denote a statistically significant difference (p < 0.05) compared to H4 data.

As *RSU-1* has been previously reported to be overexpressed in metastatic breast cancer samples as well as highly invasive breast cancer and hepatocellular carcinoma cell lines^19,54^, we investigated if it is differentially expressed in our glioma cell lines and if its expression is correlated with invasiveness. In that regard, we first tested the expression of *RSU-1* at the mRNA level. Real-Time PCR was performed for the *RSU-1* gene using actin as housekeeping gene and H4 cell line as calibrator (Fig. 3A). Our results showed that the more aggressive A172 and U87-MG cell lines overexpressed *RSU-1* compared to the less aggressive H4 and SW1088 cell lines. Real-Time PCR results were also validated by immunoblotting (Fig. 3B,C, note lanes 3 and 4 compared to lanes 1 and 2, as well as Supplementary Fig. 4).

### Elimination of RSU-1 from glioma cells differentially affects their motility

**Figure 4:**
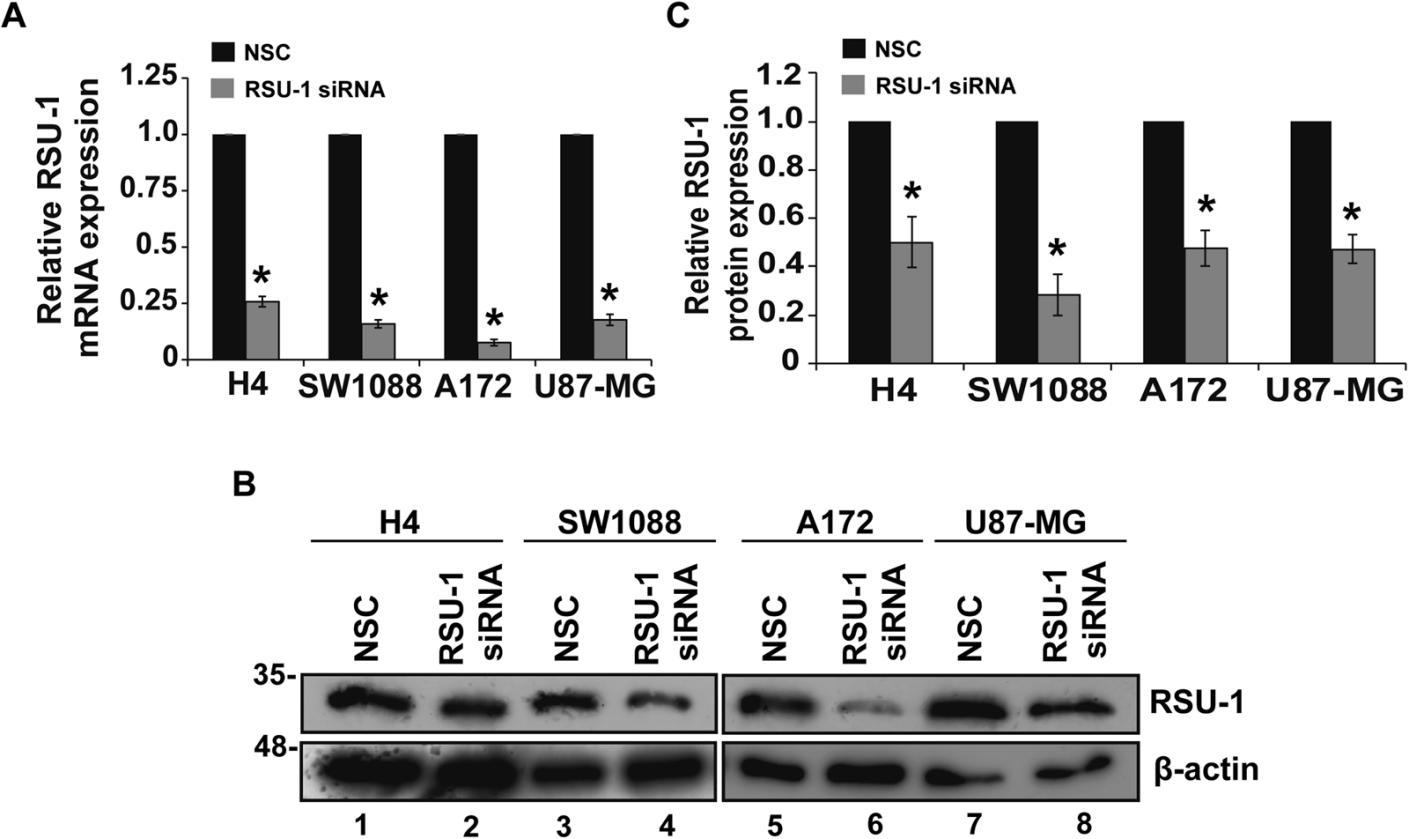
RSU-1 is effectively silenced both at the mRNA level and protein level. (**A)** Relative mRNA expression of *RSU-1* in H4, SW1088, A172 and U87-MG cells upon treatment with NSC or RSU-1 siRNA for at least 48 h. Eleven (11) independent RT-PCR experiments were performed and data were analyzed using the ΔΔCt method, having NSC treated cells as a calibrator sample for each cell line. **(B)** Representative immunoblot showing RSU1 expression at the protein level following treatment with NSC or *RSU-1* siRNA in all four glioma cell lines studied. Cropped blots are from samples run on the same gel and original pictures of the western blots are displayed in Supplementary Fig. 3. (**C)** Graph representing quantification of RSU-1 protein expression normalized to β-actin for each cell line using ImageJ software. Immunoblots from four (4) independent experiments were used for the quantification. Asterisks denote a statistically significant difference (p < 0.05) compared to NSC data.

**Figure 5:**
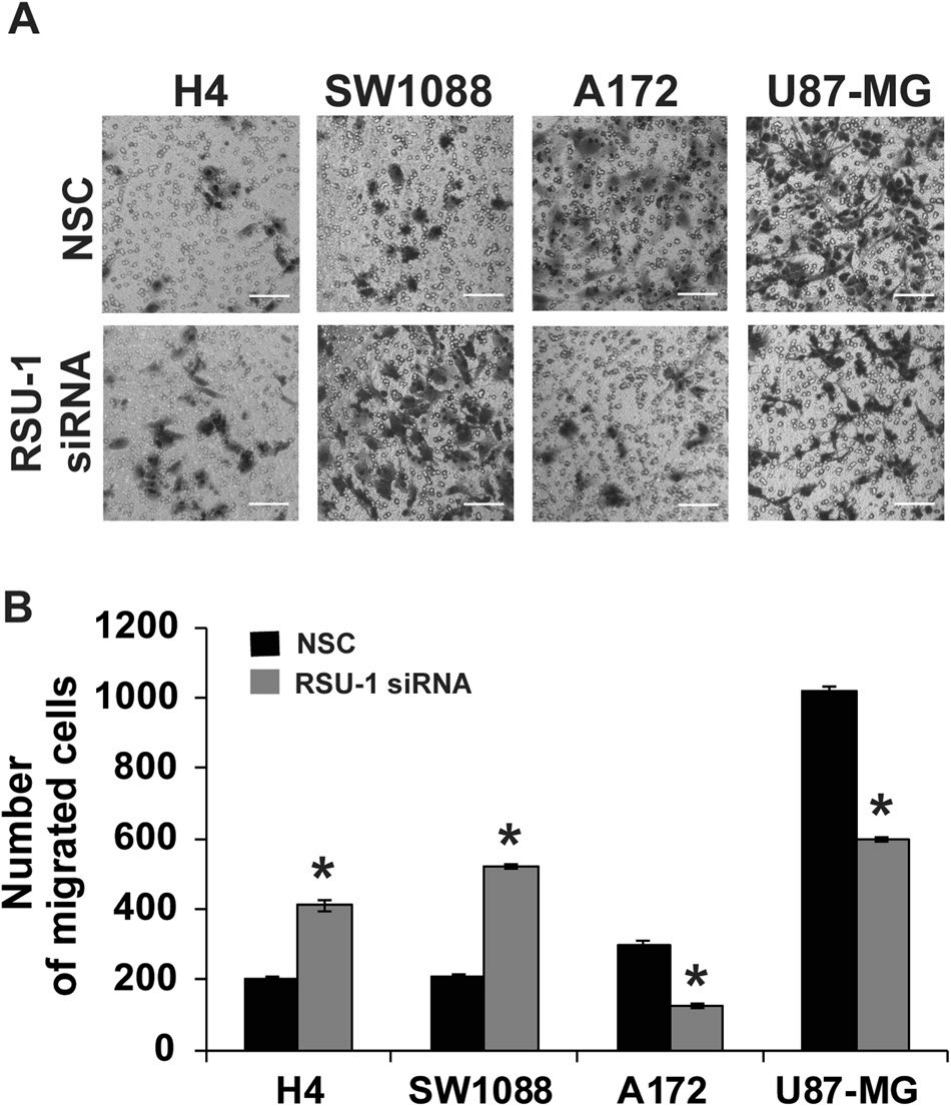
RSU-1 silencing increased migration of the non-aggressive glioma cells but decreased migration of the aggressive cells. (**A)** Representative images of a transwell migration assay that was performed for 24 h for the four glioma cell lines with NSC or RSU-1 siRNA treatment. Scale bar: 100 μm. The migrating cells were counted in nine (9) randomly chosen microscopic fields per transwell. **(B)** Total number of migrated cells compared to NSC for each cell line per transwell. Each sample was run in triplicate and three (3) independent experiments were performed. Asterisks denote a statistically significant difference (p < 0.05) compared to NSC data.

To identify the role of RSU-1 in the metastatic properties of glioma cells, it was silenced using siRNA-mediated silencing using a non-specific control siRNA (NSC) as transfection control. *RSU-1* was effectively silenced both at the mRNA (Fig. 4A) and protein (Fig. 4B,C) level. Then, transwell migration assay was performed to find out how *RSU-1* silencing affects cell motility. The number of cells that migrated through the transwell pores was counted 24 h after the addition of cells in the transwell and 48 h after siRNA transfection. Surprisingly, *RSU-1* silencing did not have the same effect in all four glioma cell lines tested. Interestingly enough though, the migratory response of cells was associated with the degree of malignancy to which we assigned them based on the results of morphological analysis, AFM measurements, soft agar growth and spheroid invasion. The least aggressive glioma cells (H4 and SW1088) exhibited increased motility following *RSU-1* silencing in contrast to the most aggressive glioma cells (A172 and U87-MG), which exhibited decreased motility after *RSU-1* silencing (Fig. 5A,B).

### RSU-1 enhances the invasion potential of aggressive glioma cells through MMP13, in contrast to non-aggressive glioma cells

**Figure 6:**
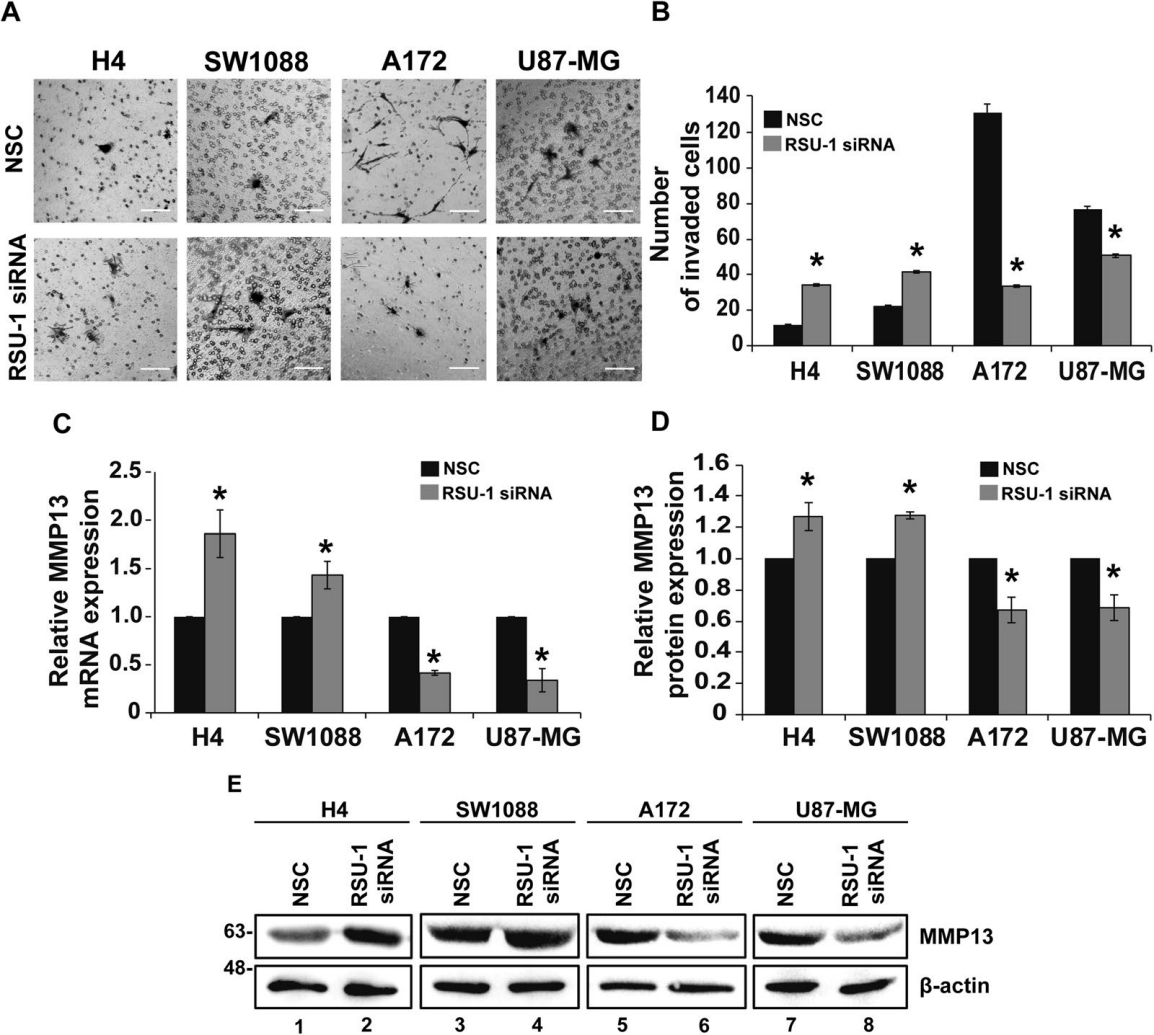
RSU-1 silencing increased invasion of non-aggressive glioma cells while decreased invasion of aggressive glioma cells through reduction in MMP-13. (**A**) Representative images of a transwell invasion assay that was performed for 24 h for the four glioma cell lines with NSC or *RSU-1* siRNA treatment. The invading cells were counted in nine (9) randomly chosen microscopic fields per transwell. Scale bar: 100 μm. (**B**) Total number of invaded cells compared to NSC for each cell line per transwell. Each sample was run in triplicate and at least three (3) independent experiments were performed. (**C**) Relative *MMP13* mRNA expression following *RSU-1* silencing for the four studying glioma cells was measured by RT-PCR and quantification was done using as NSC as the calibrator sample. (**D**) Graph representing quantification of MMP13 protein expression normalized to β-actin for each cell line following treatment with NSC or *RSU-1* siRNA in all four glioma cell lines studied. Immunoblots from three independent experiments were used for the quantification using ImageJ software. (**E**) Representative immunoblot showing MMP13 protein expression. Original pictures of the western blots are displayed in Supplementary Fig. 6. Asterisks denote a statistically significant difference (p < 0.05) compared to NSC data.

Next, transwell invasion assay was performed following *RSU-1* silencing in all glioma cell lines. In accordance with cell motility results, invasion capacity was also found to be decreased upon *RSU-1* silencing in the most aggressive glioma cells, whereas it was increased in the least aggressive cells (Fig. 6A). Figure 6B indicates the mean of the total number of invaded cells per transwell for each cell line upon *RSU-1* silencing.

Finally, we tested whether *RSU-1* silencing affects key molecules involved in matrix degradation, an important aspect of cell invasion. Thus, following *RSU-1* silencing, we tested the mRNA expression of matrix metalloproteinase13 (MMP13) a fundamental protease in cancer cell metastasis, known to be involved in collagen I degradation^55,56^ that was previously shown to be regulated by *RSU-1* silencing in breast cancer cells^20^. We found that *MMP13* mRNA expression was following a pattern identical to that of cell migration and invasion, corroborating our findings (Fig. 6C). Quantitative PCR results were validated further at the protein level by immunoblotting as shown in Fig. 6D,E.

### Rsu-1 silencing exerts its effect on glioma cell invasion through STAT6 phosphorylation regulation

**Figure 7:**
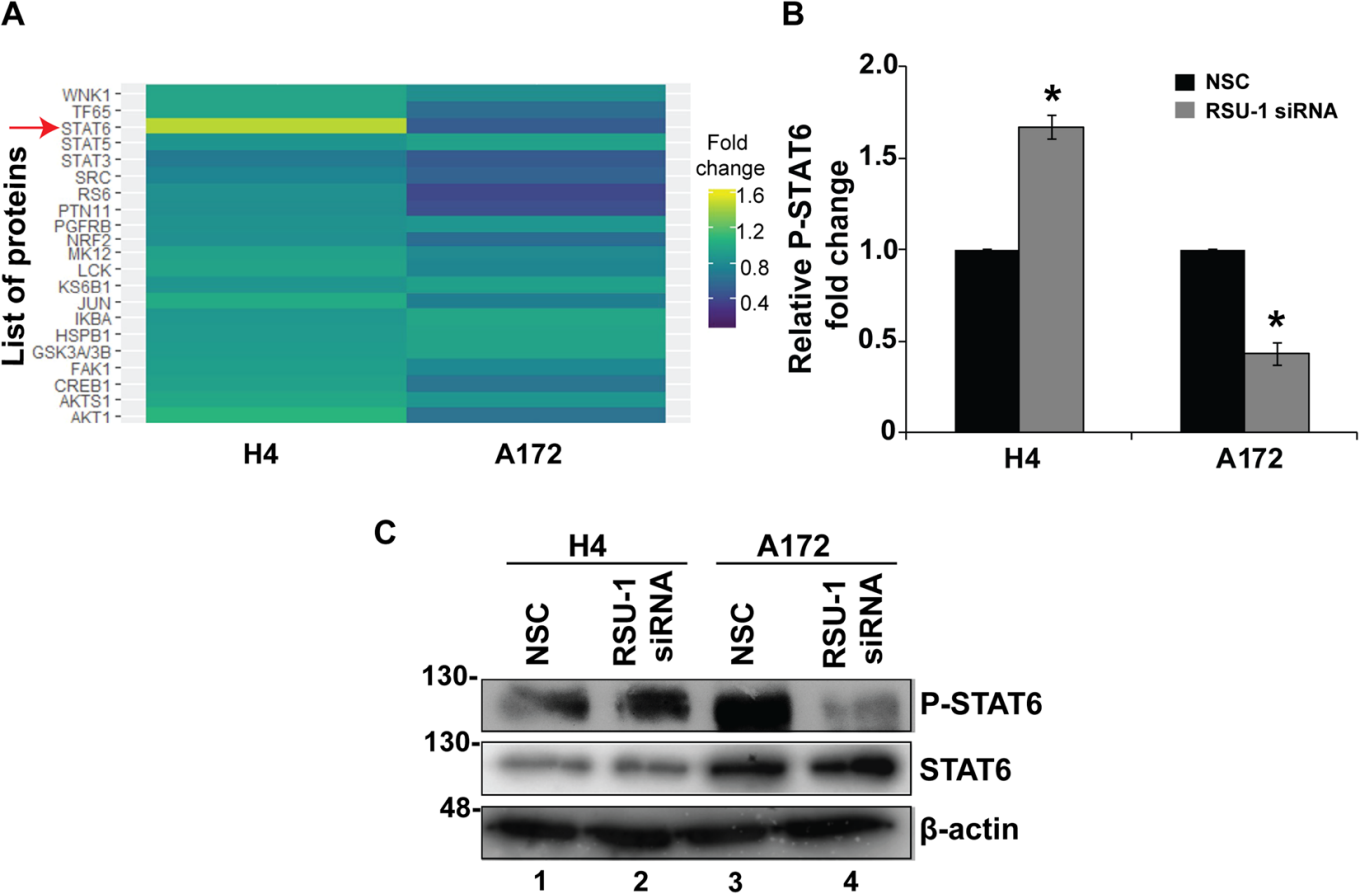
*RSU-1* silencing enhanced STAT6 phosphorylation in non-aggressive glioma cells while diminished STAT6 phosphorylation in aggressive glioma cells. (**A**) The heatmap depicts mean fold change results from the phosphoproteomic analysis performed for 21 phospho-proteins in two independent experiments between the treated (RSU-1 siRNA) and control cells (NSC siRNA) for both H4 and A172 cell lines. Red arrow indicates the most significant change in phosphorylation status upon *RSU-1* knockdown. (**B**) Quantification of the phosphoprotein analysis data for P-STAT6 following *RSU-1* silencing using NSC as the control sample. (**C**) Representative immunoblot validating the phosphorylation status of STAT6 for the same protein samples as in (**A**). Cropped blots are from samples run on the same gel and original pictures of the western blots are displayed in Supplementary Fig. 8. Asterisks denote a statistically significant difference (p < 0.05) compared to NSC.

**Figure 8:**
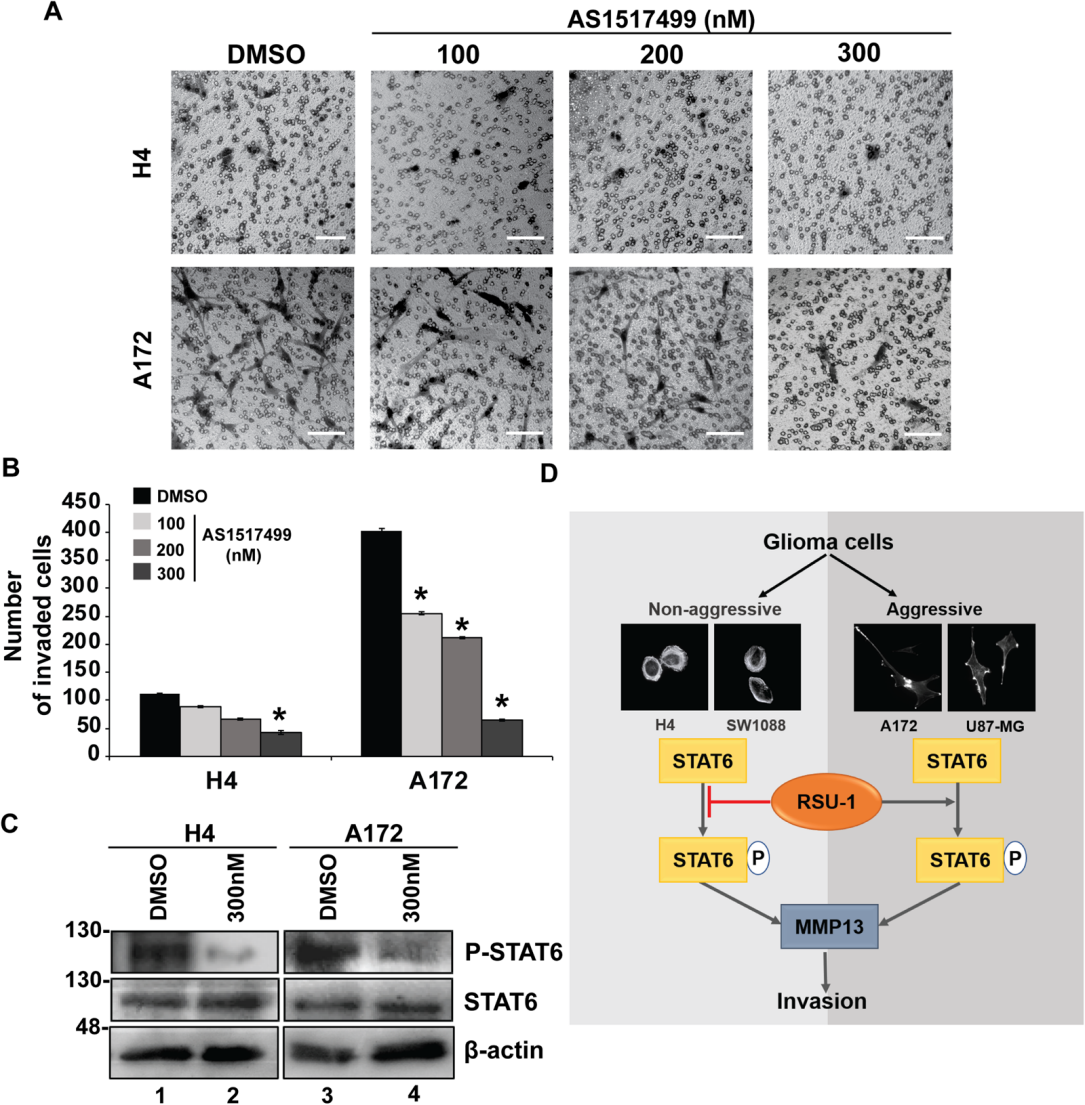
*In vitro* effects of the phospho-STAT6 inhibitor (AS1517499), in glioma cell (H4 and A172) invasion. (**A**) Representative images of transwell invasion assay performed following treatment with phospho-STAT6 inhibitor, AS1517499 (at 100, 200 or 300 nM) or DMSO for 24 h. Cells were left to invade for an additional 24 h time period with inhibitor. The invading cells were counted in nine (9) randomly chosen microscopic fields per transwell. Scale bar: 100 μm. **(B)** Total number of invaded cells compared to DMSO for each cell line per transwell. Two transwells were included per sample and at least two (2) independent experiments were performed. **(C)** Representative images of Western blot results of STAT6 phosphorylation in glioma cell lines (H4 and A172) following treatment with DMSO or 300 nM of AS1517499. Original pictures of the western blots are displayed in Supplementary Fig. 10. Asterisks denote a statistically significant difference (p < 0.05) compared to DMSO. **(D)** Schematic diagram illustrating the significant findings of this work.

Although the connection between cell invasion and MMP13 expression is well-established, we investigated whether other signaling molecules are mediating the effect of *RSU-1* silencing on cell invasion. To that regard, we selected the least invasive (H4) and one of the most invasive (A172) cells from the glioma cell panel, treated them with NSC or *RSU-1* siRNA and analyzed their protein expression using a Multiplexed Assay specifically designed to detect the 21 most influential phospho-proteins. Analysis of the Multiplex assay (Fig. 7A) showed that only Signal Transducer and Activator of Transcription6 (STAT6) exhibited changes in phosphorylation that were consistent with the observed invasion pattern as well as with recently published data showing STAT6 to promote invasion in glioma cells^57^. More specifically, the least invasive cells (H4) treated with *RSU-1* siRNA had an increased level of phospho-STAT6 and a more invasive potential (Fig. 7B), whereas the more invasive cells (A172) treated with *RSU-1* siRNA exhibited decreased phospho-STAT6 levels and a less invasive capacity (Fig. 7A,B). The multiplex assay results were validated further by immunoblotting for H4 and A172 cell lines (Fig. 7C) as well as SW1088 and U87-MG cell lines (Supplementary Fig. 7). To test whether STAT-6 phosphorylation is crucially involved in glioma cell invasion, we inhibited it in H4 and A172 cells by varying the concentration (100, 200 & 300 nM) of the AS1517499 inhibitor, and we found that their invasive capacity was reduced upon inhibition of STAT-6 phosphorylation in a dose-dependent manner in both cell lines (Fig. 8A,B). These results were then validated in SW1088 and U87-MG cells using only the optimum concentration of the AS1517499 (300 nM) as shown in Supplementary Fig. 11.

## Discussion

In the current study, we used four glioma cell lines, namely H4, SW1088, U87-MG and A172^48–50,58^ and we first characterized them based on their morphology, actin cytoskeleton organization, stiffness and invasion capacity. Our results show that A172 and U87-MG cells, which cause malignant tumors, were more elongated (Fig. 1A), exhibited well-organized actin fibers, increased invasion in transwells and in collagen-embedded spheroids (Fig. 2A, Supplementary Fig. 1) and increased formation of colonies in soft agar (Fig. 2B) showing metastatic abilities and capability for anchorage independent growth, respectively. Conversely, H4 and SW1088 cells were less elongated (Figs. 1A,D), presented random orientation of actin stress fibers (Fig. 1C), reduced invasion (Fig. 2A) and inability to grow in soft agar (Fig. 2B). Moreover, AFM measurements demonstrated that A172 and U87-MG cells exhibited statistically significant lower Young’s modulus than H4 and SW1088, indicating that malignant glioma cells are softer than H4 and SW1088 a property that enables them to migrate faster (Fig. 1E). These findings are consistent with the results of pertinent studies in other cancer cell lines, which also showed that highly aggressive cancer cells are generally softer than non-malignant cells^53,59,60^. Interesting, the ratio of the Young’s modulus value of the less invasive cells (non-tumorigenic, H4) to the Young’s value of the other cell lines are: H4/SW1088 = 0.73, H4/A172 = 1.58, H4/U87-MG = 1.53. These results are similar to the results found in the literature^53,61^ and especially to the ratio (1.4–1.8) between healthy and breast cancer cells where similar spherical probes were used^62^. We then tested the expression of RSU-1, a FA protein that was previously shown to be implicated in breast cancer cell invasion promoting breast cancer cell metastasis, introducing RSU-1 as a potential metastasis marker^20^. We found here that *RSU-1* is upregulated in cell lines exhibiting higher invasion capacity (A172 and U87-MG) compared to the less invasive cells (H4 and SW1088) both at the mRNA (Fig. 3A) and protein level (Fig. 3B,C). Interestingly, we showed for the first time that *RSU-1* silencing has an opposite effect on glioma cell line invasion depending on whether the cell line is aggressive or not (Fig. 6A,B). More specifically, *RSU-1* silencing in A172 and U87-MG cell lines inhibited their invasion, whereas it promoted invasion of H4 and SW1088 cells. Interestingly, *MMP13* expression followed an almost identical pattern (Fig. 6C,D). Finally, we also showed through multiplex analysis of phospho-proteins that STAT6 phosphorylation was increased in the H4 cell line upon *RSU-1* silencing in contrast to A172 cells in which STAT6 phosphorylation was decreased (Fig. 7A,B). Multiplex results were validated by immunoblotting further confirming the involvement of STAT6 in glioma cell invasion (Fig. 7C) as demonstrated in other cancer types^63^. Interestingly, we also found that the invasive capacity of H4 and A172 glioma cells was decreased following inhibition of STAT6 phosphorylation in a dose-dependent manner (Fig. 8A,B). This result is in accordance with other previously published studies, showing that STAT-6 promotes glioma cells invasion^57^, although this is the first time that *RSU-1* is being associated with STAT6 regulation as shown in Fig. 7D.

In conclusion, the present study provides the first evidence that RSU-1 has distinct roles in glioma cell invasion depending on the cells’ aggressiveness. In fact, our finding that depletion of *RSU-1* from the highly invasive A172 cells -that normally express RSU-1 in high levels- inhibits cell invasion, whereas depletion of *RSU-1* from the non-invasive H4 cells -that normally express RSU-1 at minimal levels-enhances cell invasion indicates that there exists a type of regulation that is level-dependent. This is reminiscent of other cases in which the expression level of a FA protein is correlated with differential regulation of cell migration^64^, and definitely warrants further investigation. Further investigation is, of course, warranted in order to decipher the exact mechanism of action of RSU-1 in gliomas. Moreover, validation of the current findings in human glioma patients, especially of varying tumor grade would be rather valuable and might render RSU-1 a predictor of gliomas progression potentially contributing to the development of novel therapeutic interventions targeting it. Hence, patients with elevated *RSU-1* expression would be expected to have aggressive gliomas and would benefit from a treatment that includes blocking *RSU-1* whereas patients with reduced *RSU-1* expression would be expected to have less aggressive tumors and would thus benefit from a conventional treatment.

## Supplementary information

Supplementary Figure
