# Supplementary material for: Ras suppressor-1 (RSU-1) promotes cell invasion in aggressive glioma cells and inhibits it in non-aggressive cells through STAT6 phospho-regulation
Source: Sci Rep. 2019 May 23;9:7782. doi: 10.1038/s41598-019-44200-8 (PMC6533309; doi:10.1038/s41598-019-44200-8)
Supplement: Supplementary file 1 — Supplementary Figure [file 41598_2019_44200_MOESM1_ESM.pdf]

Ras suppressor-1 (RSU-1) promotes cell invasion in aggressive glioma cells and inhibits it in non-aggressive cells through STAT6 phospho-regulation

MARIA LOUCA[1], ANDREAS STYLIANOU[1], ANGELIKI MINIA[2], VAIA PLIAKA[2], LEONIDAS G ALEXOPOULOS[2,3], VASILIKI GKRETSI[4*] and TRIANTAFYLLOS STYLIANOPOULOS[1*]

[1]Cancer Biophysics Laboratory, Department of Mechanical and Manufacturing Engineering, University of Cyprus, Nicosia, Cyprus.

[2]ProtATonce Ltd., Athens, Greece

[3]Department of Mechanical Engineering, National Technical University of Athens, Athens, Greece

[4]Biomedical Sciences Program, Department of Life Sciences, School of Sciences, European University Cyprus, Nicosia, Cyprus

*Corresponding authors

Address correspondence to:

Triantafyllos Stylianopoulos, Ph.D.

Cancer Biophysics Laboratory, Department of Mechanical and Manufacturing Engineering

University of Cyprus, P.O. Box 20537, Nicosia, 1678, Cyprus, tel: +357 2289 2238, fax: +357 2289 5081,

E-mail: tstylian@ucy.ac.cy

or

Vasiliki Gkretsi, Ph.D,

Biomedical Sciences Program, Department of Life Sciences, School of Sciences, European University Cyprus, 6 Diogenous Street, Egkomi, 1516 Nicosia, Cyprus

E-mail: vasso.gkretsi@gmail.com.

Supplementary Information

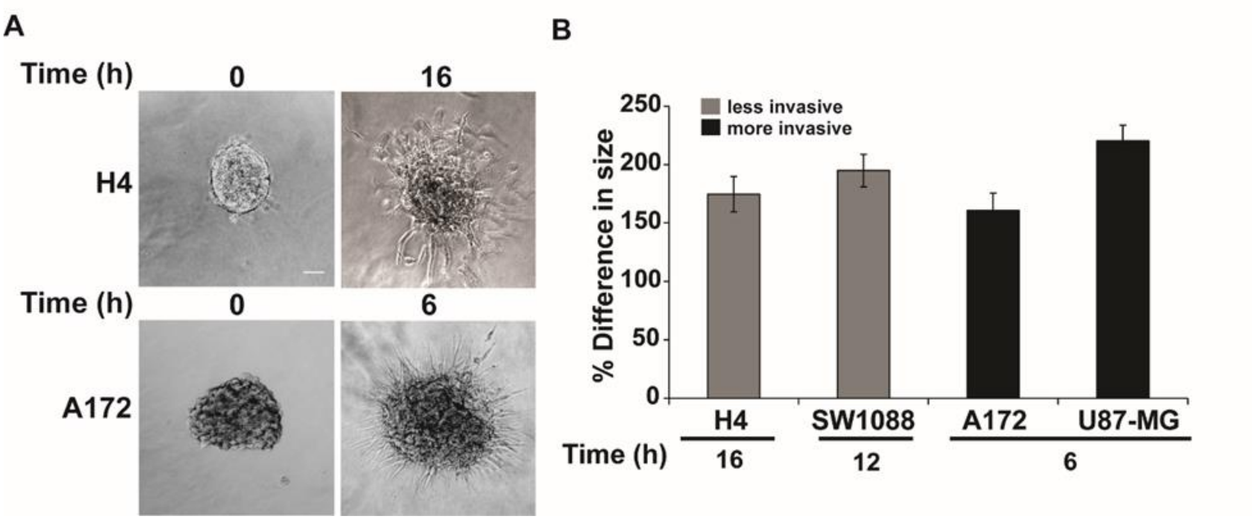

Supplementary Figure 1: Tumor spheroid invasion assay in collagen gels. Spheroids (in average n=15 spheroids per cell line) were embedded in 1mg/ml collagen I gel and left to invade through the gel for different time periods depending on the aggressiveness of each cell line. **(A)** Representative images of H4 (least invasive) and A172 (most invasive) cells for 16h and 6 h, respectively. **(B)** The percentage of tumor spheroid invasion in each case was assessed by measuring the difference of each spheroid size ((major+minor axis)/2) within the corresponding hours following placement of the spheroid in the collagen gel (time zero).

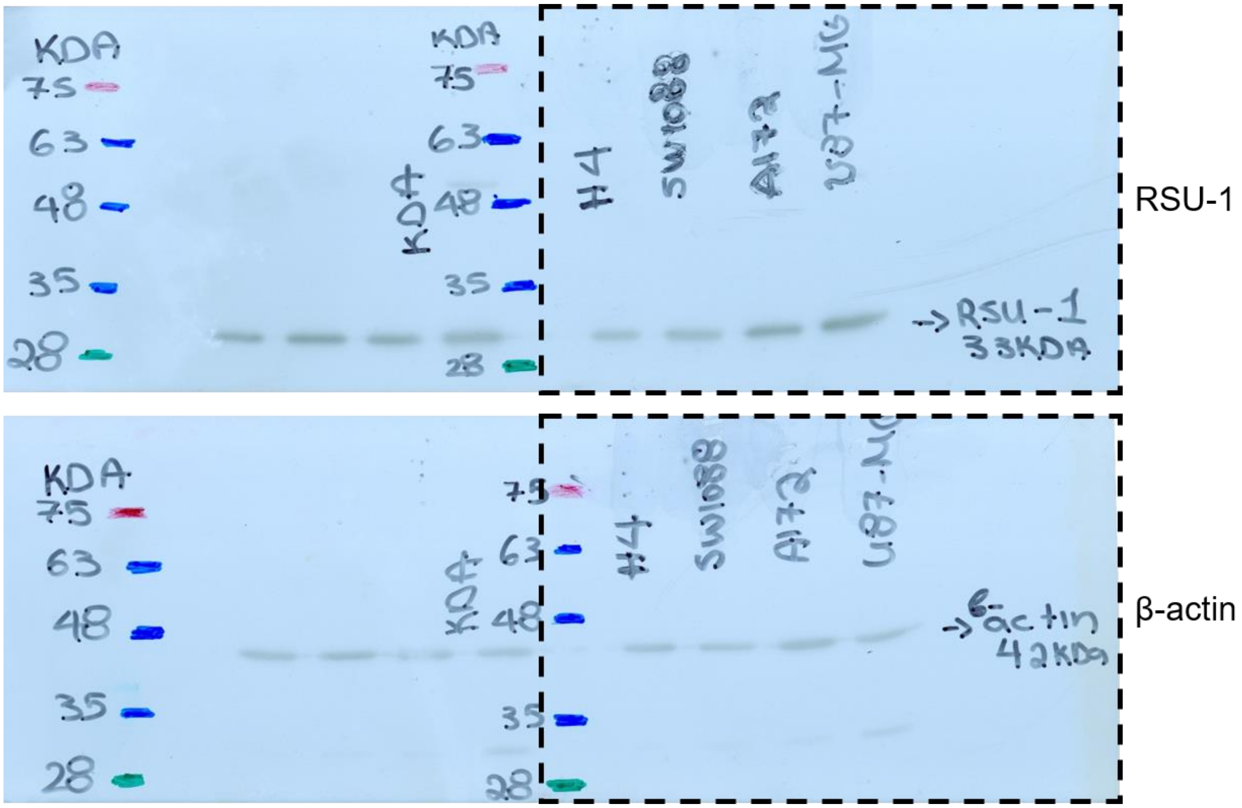

Supplementary Figure 2: Original pictures of the western blot for Figure 3B

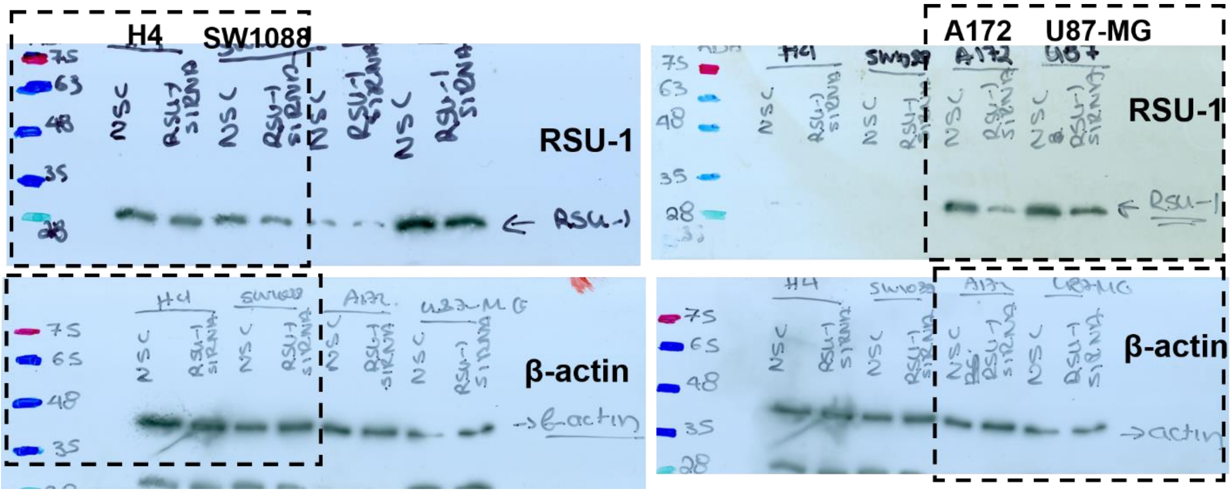

Supplementary Figure 3: Original pictures of the western blots for Figure 4B

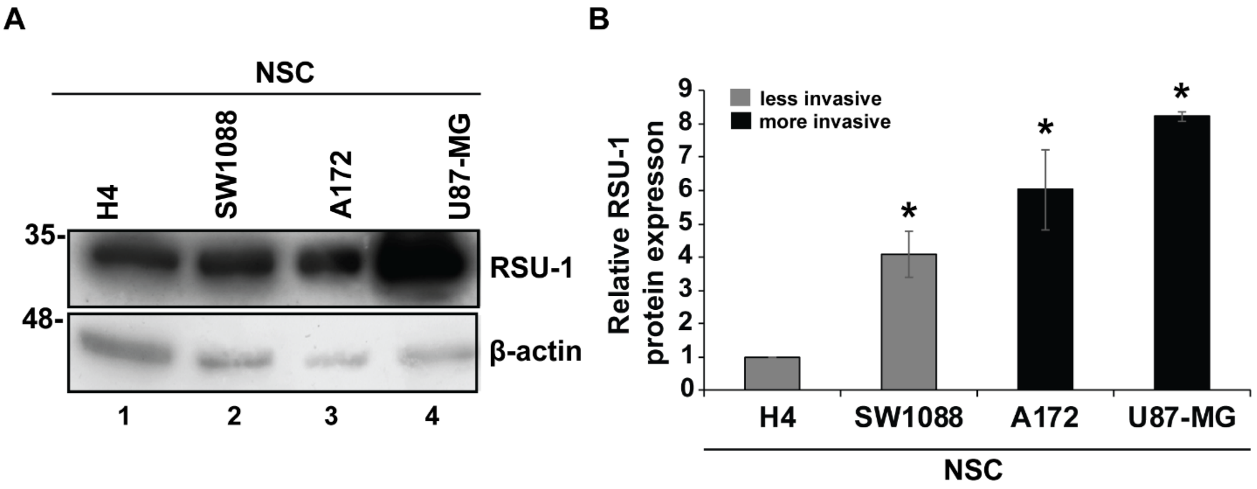

Supplementary Figure 4: (A) Representative immune blot using the same samples as in Figure 3 that include whole cell lysates from glioma cells treated with NSC siRNA. This figure confirms that RSU-1 expression level is elevated in more aggressive compared to less aggressive glioma cells. Cropped blots are from samples run on the same gel while original pictures of the western blots are displayed in Supplementary Figure 5. **(B)** Graph showing the quantification of RSU-1 protein expression by ImageJ software from two immunoblots. Asterisks denote a statistically significant difference ($p<0.05$) compared to H4 cells.

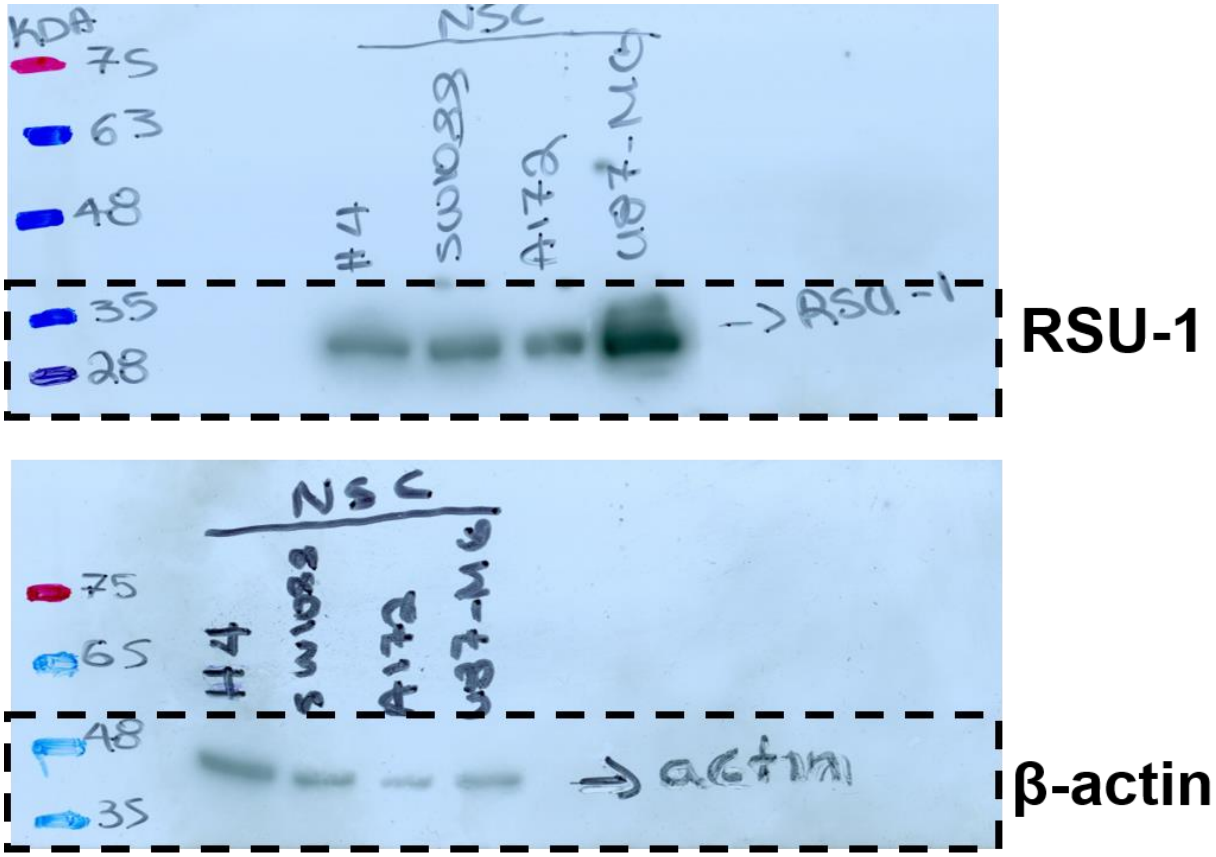

Supplementary Figure 5: Original pictures of the western blot for Supplementary Figure 4

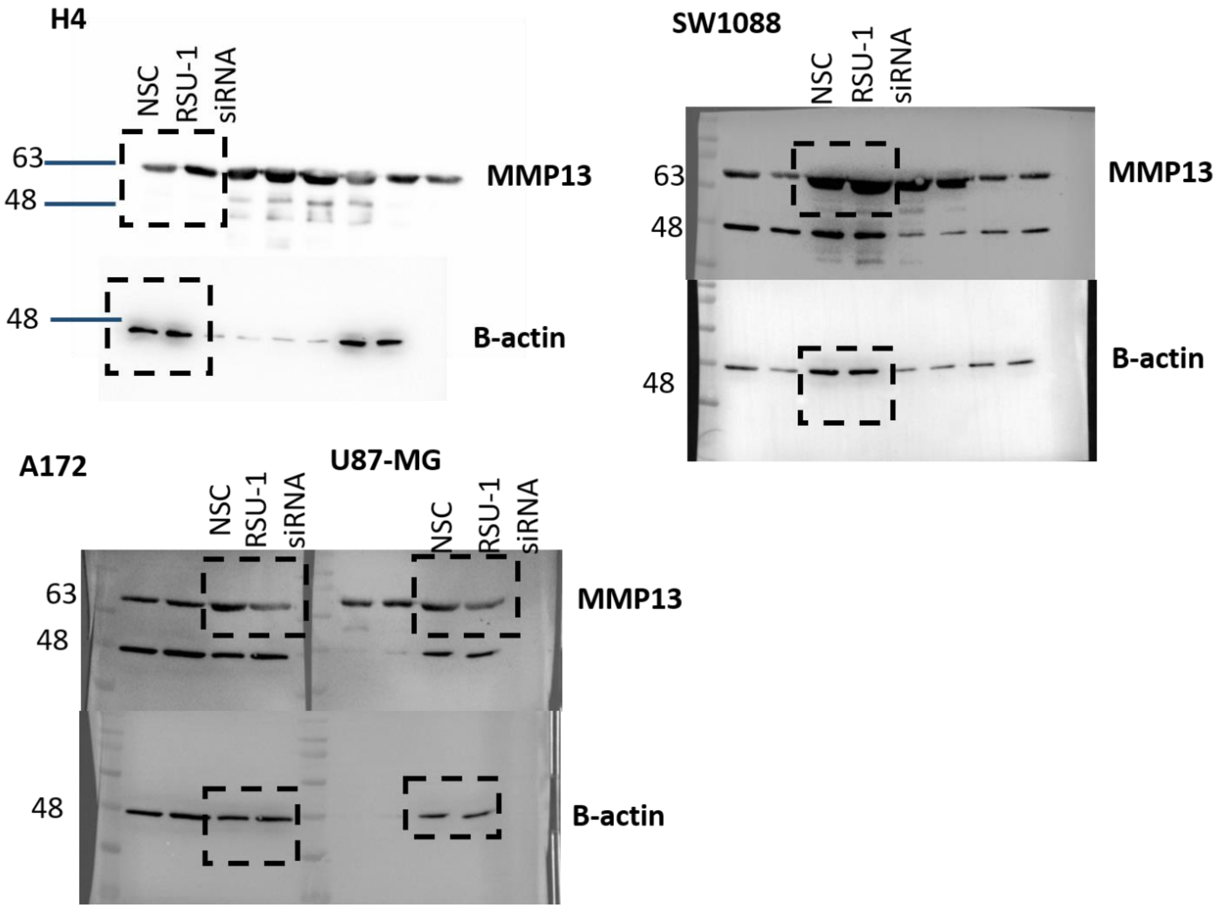

Supplementary Figure 6: Original pictures of the western blot for Figure 6D

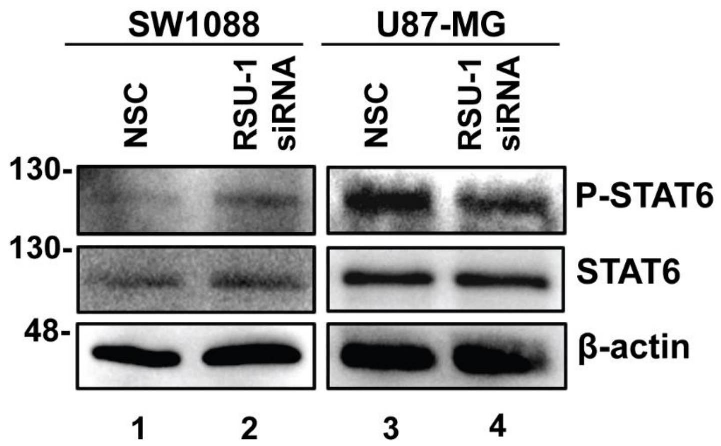

Supplementary Figure 7: Representative immunoblot showing the phosphorylation status of STAT6 in SW1088 cell line (upregulation) and in U87-MG cell line (downregulation) after *RSU-1* silencing. Original pictures of the western blot are displayed in Supplementary Figure 9.

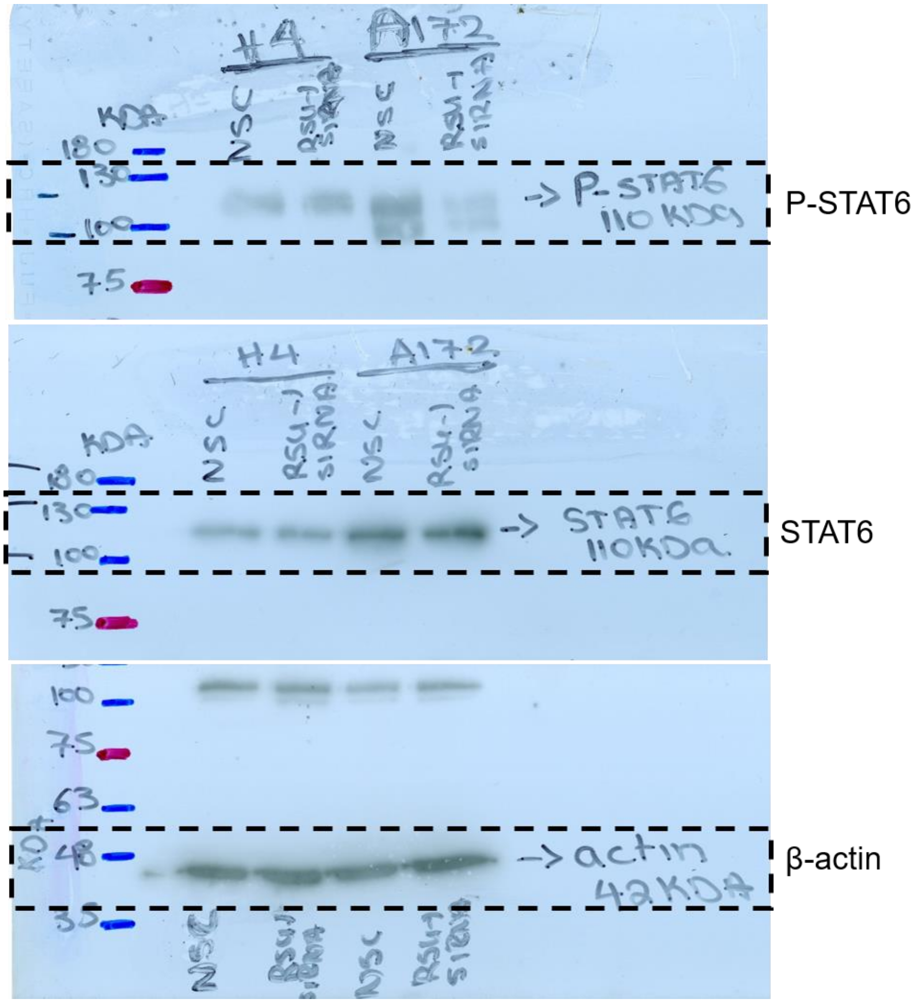

Supplementary Figure 8: Original pictures of the western blot for Figure 7C

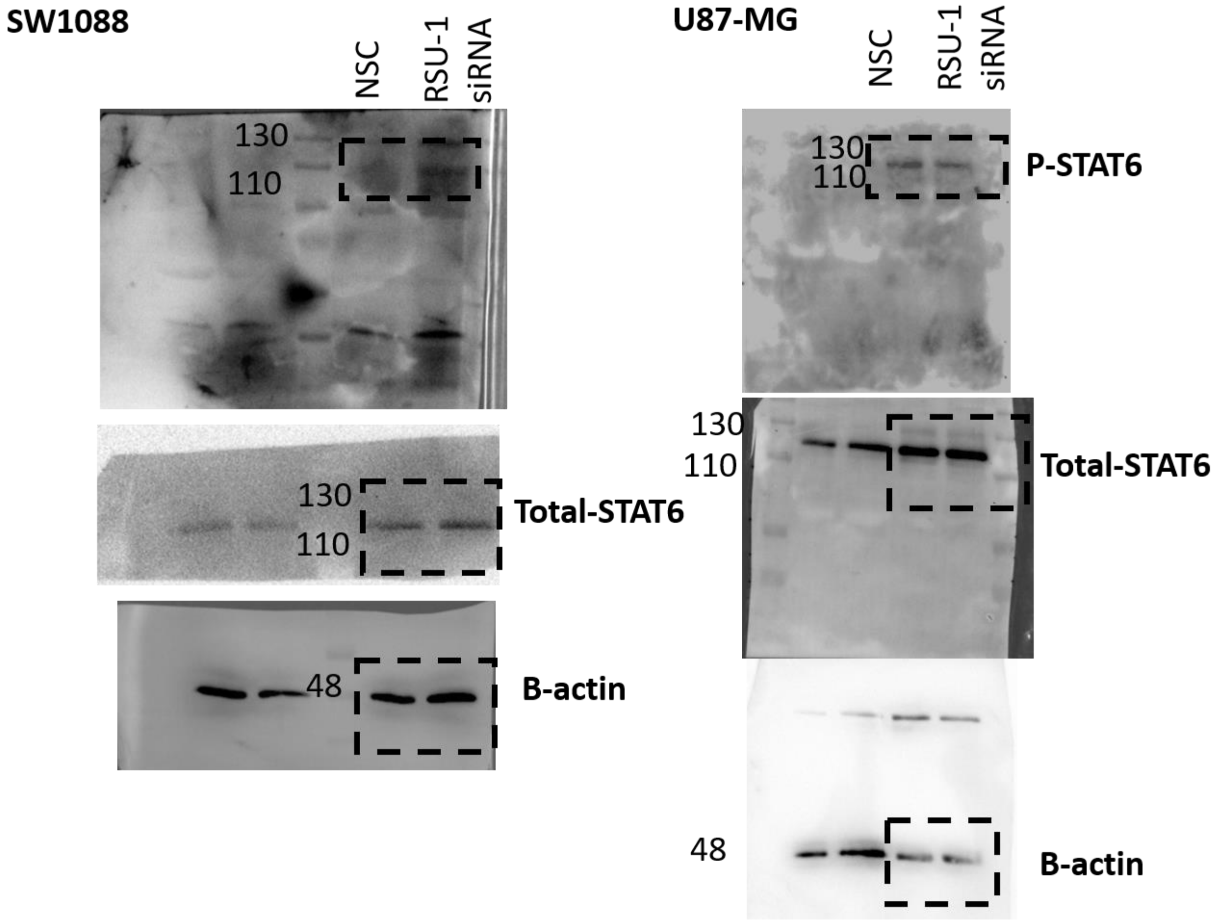

Supplementary Figure 9: Original pictures of the western blot for Supplementary Figure 7

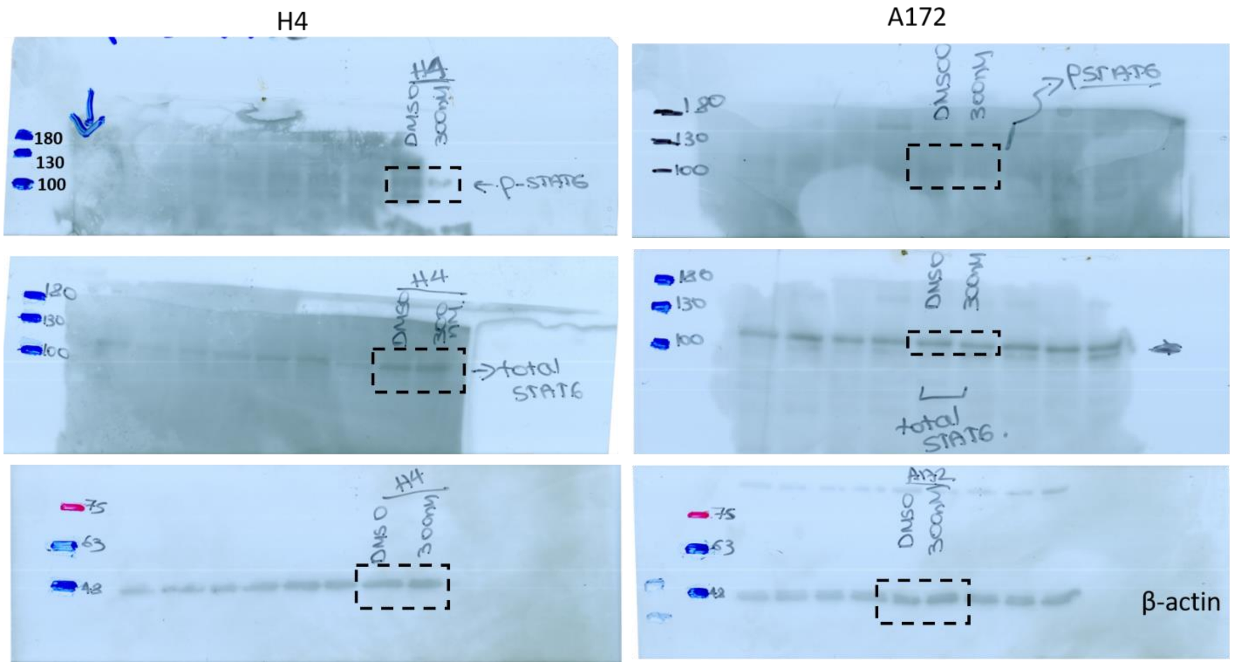

Supplementary Figure 10: Original pictures of the western blot for Figure 8

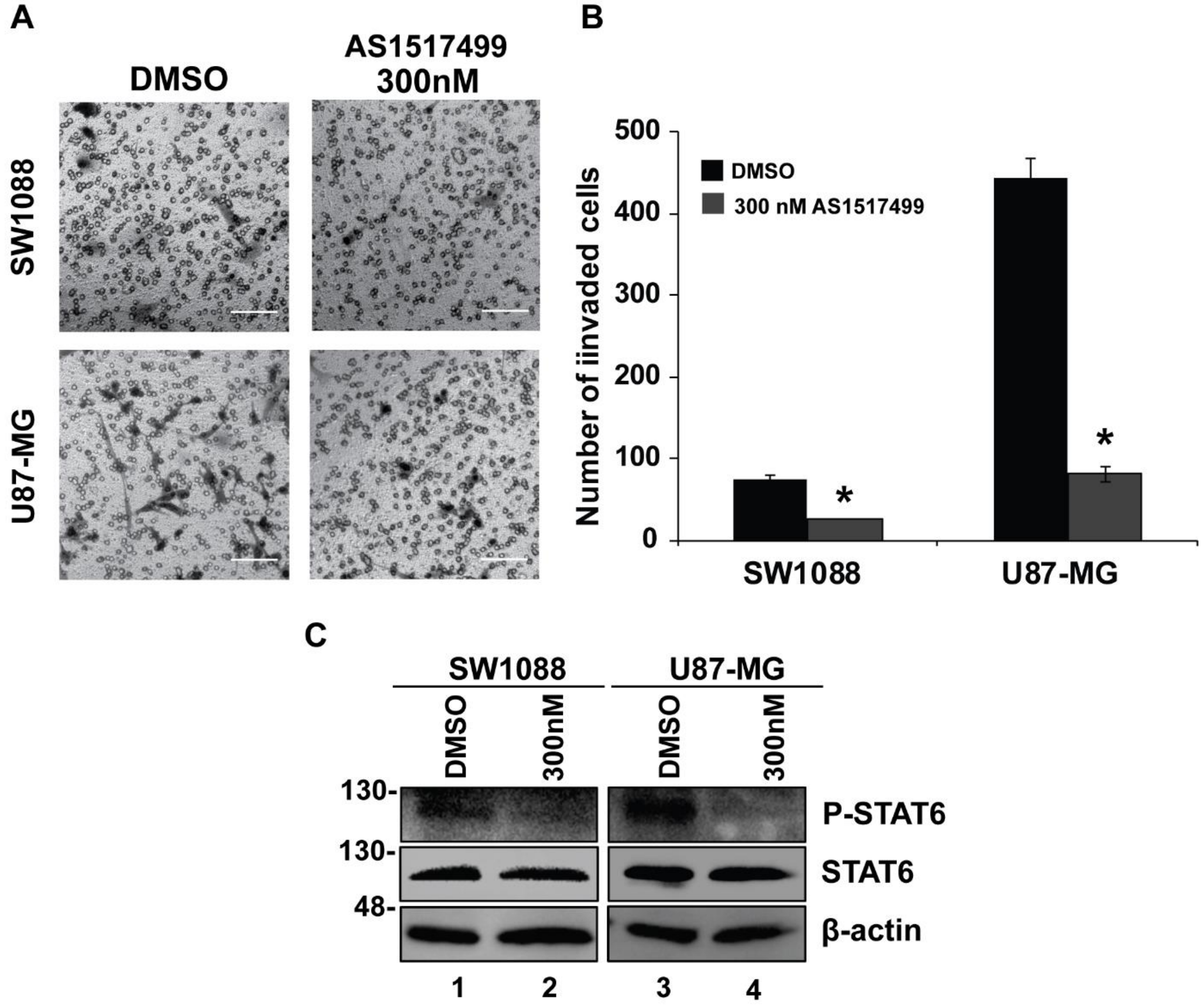

Supplementary Figure 11: The effect of the phospho-STAT6 inhibitor (AS1517499) in SW1088 and U87-MG cell invasion. **(A)** Representative images of transwell invasion assay performed following treatment with phosphor-STAT6 inhibitor, AS1517499 (300nM) or DMSO for 24h. Cells were left to invade for an additional 24h time period in the presence of the inhibitor. The invading cells were counted in nine (9) randomly selected microscope fields per transwell. Scale bar: 100μm. **(B)** Total number of invaded cells compared to DMSO for each cell line per transwell. Three transwells were included per sample. **(C)** Representative images of western blot results of STAT6 phosphorylation in SW1088 and U87-MG cells following treatment with DMSO or 300nM of AS1517499. Original pictures of the western blots are displayed in Supplementary Figure 12. Asterisks denote a statistically significant difference ($p<0.05$) compared to DMSO.

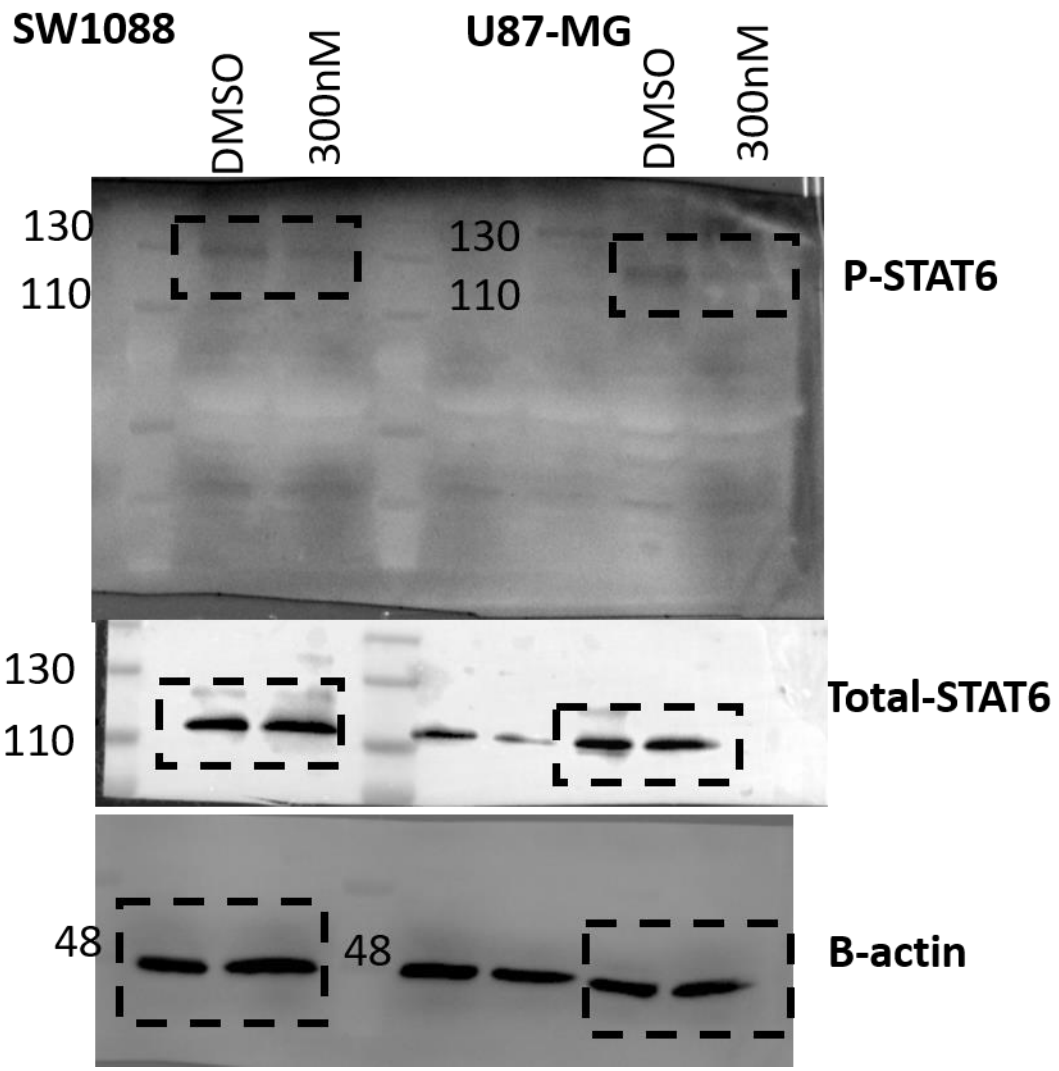

Supplementary Figure 12: Original pictures of the western blot for Supplement Figure 11
